# Coordinated multi-sectoral efforts needed to address the COVID-19 pandemic: lessons from China and the United States

**DOI:** 10.1186/s41256-020-00150-7

**Published:** 2020-05-07

**Authors:** Zhuo Chen, Cong Cao, Gonghuan Yang

**Affiliations:** 1 College of Public Health, University of Georgia, Athens, GA USA; 2 University of Nottingham Ningbo China, Ningbo, Zhejiang China; 3 Institute of Basic Medical Sciences, Chinese Academy of Medical Sciences; School of Basic Medicine, Peking Union Medical College, Beijing, China

**Keywords:** COVID-19, Public health system, Public health surveillance, Multi-sectoral efforts

## Abstract

The COVID-19 pandemic has caused staggering human and economic costs. We outline four key lessons learned from efforts to address the pandemic in China and the US. First, effective surveillance, reporting, and contact tracing are needed to contain an epidemic at its emergence and to mitigate its impact at a later stage. Second, multi-sectoral efforts to offer incentives for those with no or minor symptoms to seek care and to quarantine themselves are critical, which would need concerted efforts from payers, providers, and public health. Third, sustained and routine prevention efforts involving both the public and the health systems will prove to be useful in times of a pandemic. Fourth, a strong public health system is essential and will be appreciated at times of urgency. Concerted multi-sectoral efforts are required to address COVID-19 pandemic with strong leadership from the public health sector.

## Background

In December 2019, an outbreak of a novel coronavirus pneumonia (COVID-19) occurred in the City of Wuhan, China. As of April 22, 2020, the WHO reported 2,397,217 confirmed cases and 162,956 deaths worldwide [1]. COVID-19 is an acute respiratory disease with an estimated case fatality rate of 1–3% and an R_0_ of approximately 2.2 [2, 3]. Additional challenges in containing COVID-19 include its presumed asymptomatic carrier transmission and an incubation period ranging from 2 to 14 days [4].

Although the epidemic appears to have been under control in China, its spread outside of China led to the declaration of a worldwide pandemic by the World Health Organization on March 11, 2020. In addition to the growing number of deaths associated with COVID-19, the pandemic has put millions of people in full or partial quarantine, disrupted commerce, and caused meltdowns of the global financial market, with no clear end in sight at this moment. The rapidly changing situation warrants concerted efforts from governments around the globe. A viewpoint published 10 years after the 2002–2003 Severe Acute Respiratory Syndrome (SARS) outbreak highlighted the need for enhanced disease and symptom surveillance systems, effective infection control in all healthcare settings, and a central focus of public health for coordination and leadership with delegated responsibility and authority, among other things [5]. Unfortunately, the calls still stand true today for China, and possibly for the US and the rest of the world as well. In this commentary, we reemphasize the importance of the multi-sectoral collaboration between public health and healthcare provider, payer, and sectors beyond healthcare with lessons learned from efforts in China and the US to address the COVID-19 pandemic.

## Surveillance, reporting, and contact tracing

Detection and prevention are key to controlling epidemics. In the aftermath of the SARS epidemics, China created an electronic infectious disease reporting system that collects individual case data with unified reporting card to the national Chinese Center for Disease Control and Prevention. Evidence indicated a delay in reporting of the early COVID-19 cases in the system, alluding to a lack of training of hospital staff and deficiencies in enforcing the reporting protocols, an important lesson for public health systems elsewhere. Early identification of cases and their close contacts are key in suppressing transmission of infectious diseases. Along with social distancing, massive efforts in contact tracing have paid off in containing the epidemic in the Chinese City of Ningbo [6].

However, in COVID-19 hotspots in the US, contact tracing is mostly on backburner because of limited resources and rapidly rising number of cases at this time. As asymptomatic patients appear to be as contagious as those who have symptoms, it is critical to identify close contacts of COVID-19 patients and to implement effective self-isolation and quarantine [6]. Hotspots in the US, including the New York City, have chosen not to test patients with mild symptoms, potentially leaving a path of further transmission.

## Payers, providers, and public health

Successful containment of an emerging infectious disease requires aligning incentives between healthcare payers and providers to protect public’s health. Because COVID-19 is highly contagious, subsidized testing and treatment of its patients should be made available in time. For example, China’s National Healthcare Security Administration stipulated coverage of treatments for COVID-19 on January 22, 2020, waiving the requirements for patients to seek treatments at preferred providers and excluding COVID-19 expenses from the capitation payments for providers [7].

The US government declared COVID-19 testing as an essential health benefit, for which insurers are required by the Patient Protection and Affordable Care Act (ACA) to cover the tests. State governments followed suit to direct insurers to waive out-of-pocket costs of COVID-19 tests. However, disadvantaged populations deserve special attention. In the US, 28 million people do not have health insurance [8]. They would not be able to consult their family physician before going out for testing of COVID-19. In addition, although the U.S. Citizen and Immigration Services has been made clear that screening, testing, and treatment of COVID-19 are not considered as “public charge,” undocumented immigrants may be deterred to seek public assistance [9]. Spanish-speaking population was found to be at greater risk of exposure to H1N1 during the 2008–2009 outbreak. They, along with African Americans who have high rates of obesity and diabetes, have suffered high rates of COVID-19 infection and mortality. The epidemic will be contained only with quick and decisive actions to protect the vulnerable populations.

Of note is that the private sector has also played a critical role in containing the pandemic. Manufacturers, particularly those in China, have ramped up production of face masks and personal protective equipment to meet the growing demand from the healthcare providers and the public. Government directives on social distancing and shelter-in-place have met little or no resistance in China while they have encountered sporadic protests in the US.

## Early routine prevention to reduce COVID-19 transmission

Early routine prevention efforts including seasonal influenza vaccination may help reduce COVID-19 transmission through fewer visits of healthcare facilities during the outbreak. In the early days of the epidemic in Wuhan, many with symptoms of seasonal influenza panicked and sought screening and treatment in healthcare settings, which not only put a stress on the healthcare system but also exposed themselves to higher risks of contracting COVID-19. China’s major payers, the Healthcare Security Administrations at various level, do not cover preventive services including influenza vaccination. Preventive services in China are financed through the limited basic public health services provided at the community health services organizations. Because the savings from the preventive services would not benefit the community health services providers this could lead to a misaligned incentive.

In the US, the Advisory Committee on Immunization Practice (ACIP), an independent non-government expert panel hosted at the Centers for Disease Control and Prevention, makes routine recommendation on the effectiveness of vaccinations. Payers are mandated by the ACA to cover the vaccinations ACIP recommended without out-of-pocket costs. China and other developing countries could align such incentives to promote routine prevention efforts. Meanwhile, the US public health community will need to continue educating the public regarding the effectiveness of vaccination, particularly at a time of an outbreak.

## Public health system with strong leadership

Criticisms of public health institutions worldwide are ubiquitous. China’s healthcare and public health systems have endured the COVID-19 epidemic, with varying performances across provinces. China was able to mobilize healthcare providers from the entire country to assist healthcare facilities in Hubei Province in dealing with the overcrowding of COVID-19 patients. A key lesson is that successful efforts in epidemic detection and control demand a strong leadership of the national public health institutions and a determined political leadership and support.

Regional public health systems, as important components of the national public health system, need to be sustained. Both China’s provincial and local CDCs and US state and local public health agencies have suffered anemia of their workforces [10]. Funding dries up when there is no epidemic to worry. Ensued exodus of a competent public health workforce plagued both China and the US. Such trend jeopardizes the public health systems and should be turned around. Capacity building at the regional and local level as well as coordination between national and regional public health institutions are essential ingredients of epidemic detection and control.

## Conclusions

The COVID-19 pandemic highlights the need for concerted multi-sectoral efforts between public health and healthcare, between payers and providers, and between public health agencies at the national level and regional or local levels. Leadership and collaboration from national and regional leaders, public health institutes, and providers are in urgent need to address this unprecedented global public health challenge.

## Data Availability

Not applicable.
